# Impact of the COVID-19 pandemic on hepatitis C virus screening in provincial prisons in Montreal, Quebec, Canada

**DOI:** 10.3389/fpubh.2024.1380126

**Published:** 2024-07-23

**Authors:** Nadine Kronfli, Frederic Leone, Camille Dussault, Giovanni Miliani, Elvira Gallant, Molly Potter, Joseph Cox

**Affiliations:** ^1^Centre for Outcomes Research and Evaluation, Research Institute of the McGill University Health Centre, Montréal, QC, Canada; ^2^Department of Medicine, Division of Infectious Diseases and Chronic Viral Illness Service, McGill University Health Centre, Montréal, QC, Canada; ^3^Centre intégré universitaire de santé et de services sociaux du Nord-de-l’Île-de-Montréal – CIUSSSNIM, Montréal, QC, Canada; ^4^Ministère de la santé et des services sociaux du Québec, Montreal, QC, Canada; ^5^Faculty of Medicine and Health Sciences, Department of Epidemiology and Biostatistics, School of Population and Global Health, McGill University, Montréal, QC, Canada

**Keywords:** hepatitis C virus, screening, SARS-CoV-2, COVID-19 pandemic, prison

## Abstract

**Background:**

Little is known about the impact of the COVID-19 pandemic on hepatitis C (HCV) screening efforts in carceral settings. We explored the impact of the pandemic on HCV screening in two of Quebec’s largest provincial prisons.

**Methods:**

Retrospective data of HCV-related laboratory tests between July 2018 and February 2022 at l’Établissement de détention de Montréal (EDM) and l’Établissement de détention de Rivière-des-Prairies (EDRDP) were obtained. To examine the association between the pandemic and the number of HCV-antibody (HCV-Ab) tests, a three-level time period variable was created: pre-outbreak, outbreak, and post-outbreak. Negative binomial regression (with monthly admissions as an offset) was used to assess the change in HCV-Ab tests across time periods and by prisons. Adjusted odds ratios (aOR) with 95% confidence intervals (95% CI) were calculated.

**Results:**

A total of 1,790 HCV-Ab tests were performed; 56 (3%) were positive. Among these, 44 (79%) HCV RNA tests were performed; 23 (52%) were positive. There was a significant decrease in HCV-Ab screening at EDM during the outbreak (aOR 0.29; 95% CI 0.17–0.48) and post-outbreak (aOR 0.49; 95% CI 0.35–0.69) periods, compared to the pre-outbreak period. There was no significant change in HCV-Ab screening at EDRDP during the outbreak (aOR 0.98; 95% CI 0.49–2.11) but a significant increase in HCV-Ab screening post-outbreak (aOR 1.66; 95% CI 1.04–2.72).

**Conclusion:**

The COVID-19 pandemic negatively affected HCV screening at EDM but had minimal impact at EDRDP. To eliminate HCV from carceral settings, minimizing screening interruptions during future outbreaks and combined HCV/SARS-CoV-2 screening should be prioritized.

## Introduction

The COVID-19 pandemic placed unprecedented strain on healthcare systems worldwide, diverting financial and human resources away from national hepatitis C virus (HCV) elimination efforts, and jeopardizing the achievement of HCV elimination globally (1, 2). Canada, like many countries, has committed to the World Health Organization’s 2030 HCV elimination goals of reducing HCV incidence and mortality by 80 and 65%, respectively (3), and was on track to eliminate HCV if treatment levels were maintained between 2020 and 2030 (4). However, if treatment levels decreased – a trend that was observed in most Canadian provinces even prior to the COVID-19 pandemic (5) – Canada would fail to achieve elimination. While the impact of the COVID-19 pandemic on HCV elimination efforts has yet to be fully explored in Canada, a recent modeling study found that three provinces (Manitoba, Ontario, and Quebec) were not on track to achieve HCV elimination by 2030, underscoring the urgency of accelerating HCV diagnosis and treatment to pre-pandemic levels.

The COVID-19 pandemic further compromised access to HCV services for marginalized populations; however, few studies have explored the direct impact of the pandemic on key sub-groups driving HCV transmission. People who inject drugs (PWID) in Victoria, Australia experienced a significant reduction in HCV screening during COVID-19 lockdown restrictions (6) while PWID in Montreal, Canada experienced temporary rebounds in HCV transmission due to COVID-19 related disruptions (7). Although HCV testing decreased in two of Canada’s most populous provinces [British Columbia (8) and Ontario (9)] during the pandemic, these studies failed to explore the deleterious effects on priority at-risk groups including PWID or people who experience incarceration – two populations with considerable overlap and who have been consistently left behind in national HCV elimination efforts (10). To date, there are no published studies on the impact of the COVID-19 pandemic on HCV screening in any carceral setting worldwide, highlighting an important knowledge gap. Until the full impact of the pandemic on priority populations driving the epidemic is understood, countries risk falling behind on achieving HCV elimination by 2030.

Canadian carceral settings have witnessed several large SARS-CoV-2 outbreaks since the start of the pandemic, affecting thousands of incarcerated individuals and correctional employees (11, 12). In Canada, an estimated 38,000 people are incarcerated each day – 14,000 in federal prisons and 24,000 in provincial and territorial prisons (13), differentiated by the sentence duration. Quebec is the second largest Canadian province and the province with the highest number of people who are sentenced in provincial prison (14), meaning that decreases in HCV screening during the COVID-19 pandemic could have dramatic effects on provincial and national elimination efforts. Several preventative measures were put into place to reduce the risk of SARS-CoV-2 transmission in Quebec provincial prisons. These in turn may have had effects on overall HCV screening activities. First, the average daily incarcerated provincial population in Quebec (approximately 4,500 individuals) was reduced by a minimum of 20% (15, 16). Second, those entering provincial prison underwent a mandatory 14-day isolation period on admission, thereby reducing the number of opportunities for HCV screening during incarceration. Third, human resources were not only scarcer, but were diverted away from HCV screening. In Quebec, prison-based nurses dedicated to sexually transmitted and bloodborne infection (STBBI) screening including HCV were diverted toward SARS-CoV-2 screening in April 2020 – one month after the start of the COVID-19 pandemic in Quebec. The aim of this study was to assess the impact of these measures on HCV screening in two of Quebec’s largest provincial prisons during the COVID-19 pandemic.

## Methods

### Study design and setting

We conducted a retrospective study of all HCV-related laboratory tests (antibody and RNA) performed on incarcerated individuals between July 2018 and February 2022 at l’Établissement de detention de Montréal (EDM) and l’Établissement de detention de Rivière-des-Prairies (EDRDP). EDM and EDRDP are the only provincial prisons located in Montreal, the epicentre of the SARS-CoV-2 epidemic in Quebec. EDM is the largest provincial prison in Quebec, with a capacity of 1,400 male individuals pre-pandemic (17). During the pandemic, EDM housed approximately 800 men (11). EDRDP primarily houses individuals awaiting sentencing (on remand) and has a capacity of 541 men (17). During the pandemic, EDRDP housed approximately 350 individuals (11).

### HCV screening at EDM and EDRDP

Screening strategies for HCV at EDM and EDRDP differ (17–19). “On demand” HCV screening, whereby individuals must request HCV screening following admission, is offered to incarcerated individuals at EDM. “Risk-based” HCV screening, whereby individuals at risk of HCV infection are preferentially screened shortly following admission, is offered to incarcerated individuals at EDRDP. Tests for HCV antibody (HCV-Ab) and RNA are performed via venipuncture at both EDM and EDRDP, with estimated turnaround times of 24 h and 14 days, respectively, pre-pandemic. A positive HCV-Ab result correlates with prior exposure to HCV, while a positive HCV RNA indicates current infection. All tests are routinely sent daily to Sacré-Coeur Hospital’s OPTILAB for processing.

### Sources of data

We obtained deidentified individual-level laboratory data for all HCV-Ab and HCV RNA tests from Sacré-Coeur Hospital’s OPTILAB information system from July 2018 to February 2022. The Ministère de la sécurité publique (MSP) du Québec provided aggregated statistics on prison admissions (unpublished). The Centre intégré universitaire de santé et de services sociaux (CIUSSS) du Nord de l’Île-de-Montréal provided data on hours worked by prison-based nurses from April 2020 to February 2022 (unpublished). Although the activities performed during the hours worked could not be categorized as routine vs. COVID-related, prison-based STBBI nurses at EDM and EDRDP were directed to prioritize SARS-CoV-2 screening as of April 24, 2020 (G. Miliani, personal communication, September 5, 2023), diverting away from STBBI screening. STBBI screening in Quebec provincial prisons was re-prioritized as of January 21, 2021 (G. Miliani, personal communication, December 12, 2023). This study was approved by the McGill University Health Centre Research Ethics Board (MUHC REB #2022–8,383).

### Outcome measures

We hypothesized that HCV screening would decrease during the COVID-19 pandemic in both prisons due to diverted human resources and the implementation of emergency measures to reduce SARS-CoV-2 transmission. The primary and secondary outcome measures, total numbers of HCV-Ab and HCV RNA tests performed, respectively, were classified into three time periods: pre-outbreak, outbreak, and post-outbreak. These periods were chosen to evaluate differences in the number of tests performed before, during, and after the COVID-19 outbreaks at each prison. A prison outbreak was defined as more than five confirmed cases of SARS-CoV-2 at one time in a prison. EDM experienced two outbreaks among incarcerated individuals during the study period: April 20 to July 7, 2020 and December 23, 2020 to June 3, 2021, during which time, 266 people tested positive for SARS-CoV-2 (11). The pre-outbreak period for EDM was thus defined as prior to April 20, 2020, the outbreak periods as between April 20 and July 7, 2020 and between December 23, 2020 and June 3, 2021, and the post-outbreak periods as between July 8 and December 22, 2020 and after June 3, 2021. Conversely, EDRDP experienced only one outbreak from December 21, 2020 to April 30, 2021, during which time, 32 incarcerated individuals tested positive for SARS-CoV-2 (11). The pre-outbreak period for EDRDP was thus defined as prior to December 21, 2020, the outbreak period as between December 21, 2020 and April 30, 2021, and the post-outbreak period as after May 1, 2021. We removed duplicate (n = 127) HCV-Ab tests. We performed a sensitivity analysis whereby the outbreak period for both prisons was defined as May 2020 to January 2021, inclusively, the time when prison-based STBBI nursing efforts were directed toward SARS-CoV-2 screening.

### Statistical analysis

Descriptive statistics were calculated to show the trends in new admissions and the number (and proportion) of HCV-Ab and RNA tests performed at each prison. The locally estimated scatterplot smoothing (LOESS) method was used to depict the trend of both monthly new admissions and monthly nursing hours at each prison, with 95% confidence intervals (95% CI).

Negative binomial regression models were used to examine the change in HCV-Ab tests performed at each prison and across time periods, using the pre-outbreak period as the reference. Adjusted odds ratios (aOR) with 95% CI were calculated. We included the monthly admission rate (i.e., the number of new admissions per month) of prison entrants as an offset to account for changes in prison population over the study period. Due to limited HCV RNA data, we were unable to conduct regression analyses to examine the change in HCV RNA tests across time periods and by prisons. All analyses were performed on R statistical software (Version 4.2.3) using “geepack,” “MASS,” “lme4,” “dplyr,” “lubridate,” and “ggplot2.”

## Results

### Admissions and health services statistics

**Figure 1:**
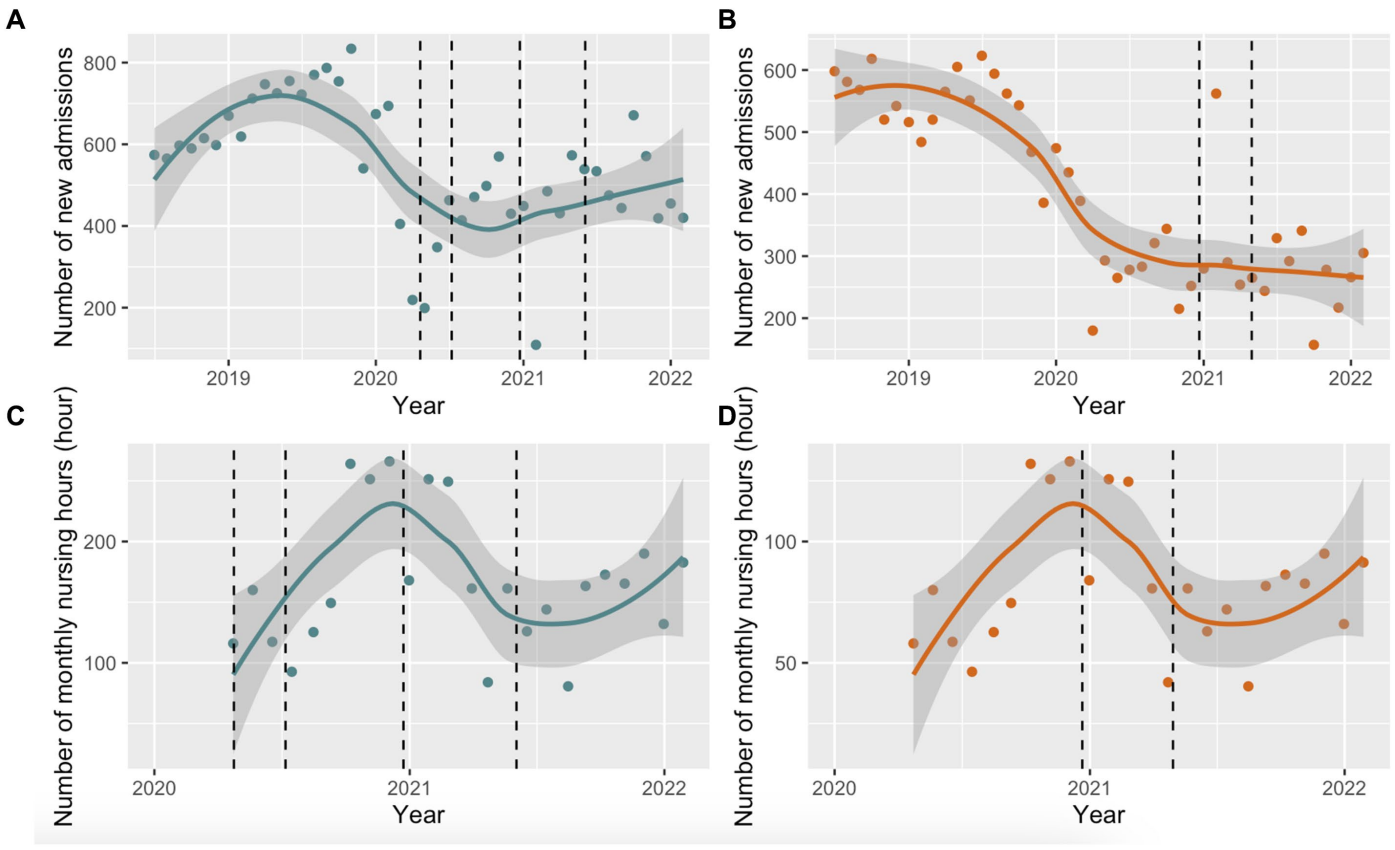
Locally estimated scatterplot smoothing (LOESS) with 95% confidence intervals (95% CI). **(A)** LOESS (95% CI) of monthly new admissions at EDM between July 2018 and February 2022. The dashed line represents the COVID-19 outbreaks at EDM (April 20 to July 7, 2020 and December 23, 2020 to June 3, 2021); **(B)** LOESS (95% CI) of monthly new admissions at EDRDP between July 2018 and February 2022. The dashed line represents the COVID-19 outbreak at EDRDP (December 21, 2020 to April 30, 2021); **(C)** LOESS (95% CI) of monthly nursing hours at EDM between April 2020 and March 2022. The dashed line represents the COVID-19 outbreaks at EDM (April 20 to July 7, 2020 and December 23, 2020 to June 3, 2021); **(D)** LOESS (95% CI) of monthly nursing hours at EDRDP between April 2020 to March 2022. The dashed line represents the COVID-19 outbreak at EDRDP (December 21, 2020 to April 30, 2021).

The number of new admissions to EDM and EDRDP during the study period are presented in Figures 1A,B, respectively. The number of new admissions to EDM peaked in mid-2019, which was followed by a reduction in admissions until the end of the same year (monthly average of ~600–700 new admissions). This was followed by a steep decline in new admissions, coinciding with the first wave of the COVID-19 pandemic in Quebec with a nadir of ~400 new admissions in September 2020, following the first COVID-19 outbreak at EDM. Thereafter, there was a modest increase in admissions until February 2022. Similar trends were observed at EDRDP with a steep decline in 2020; however, a nadir of ~250 new admissions per month was seen in 2021 (coinciding with the COVID-19 outbreak at EDRDP), with a less appreciable increase thereafter.

The CIUSSS du Nord-de-l’Île-de-Montréal estimated the number of nursing hours per month at each prison based on the number of employees. The cumulative number of nursing working hours in 2020 and 2021 at EDM were 1,710 and 2,081, respectively, and 855 and 1,041 at EDRDP, respectively. Monthly nursing hours are depicted in Figure 1C for EDM and Figure 1D for EDRDP. The number of hours worked by STBBI nurses increased linearly from April 2020, peaking at both prisons in January 2021, and decreasing linearly thereafter. There was a second increase in the number of hours worked by STBBI nurses after September 2021.

### HCV-ab and HCV RNA screening

**Figure 2:**
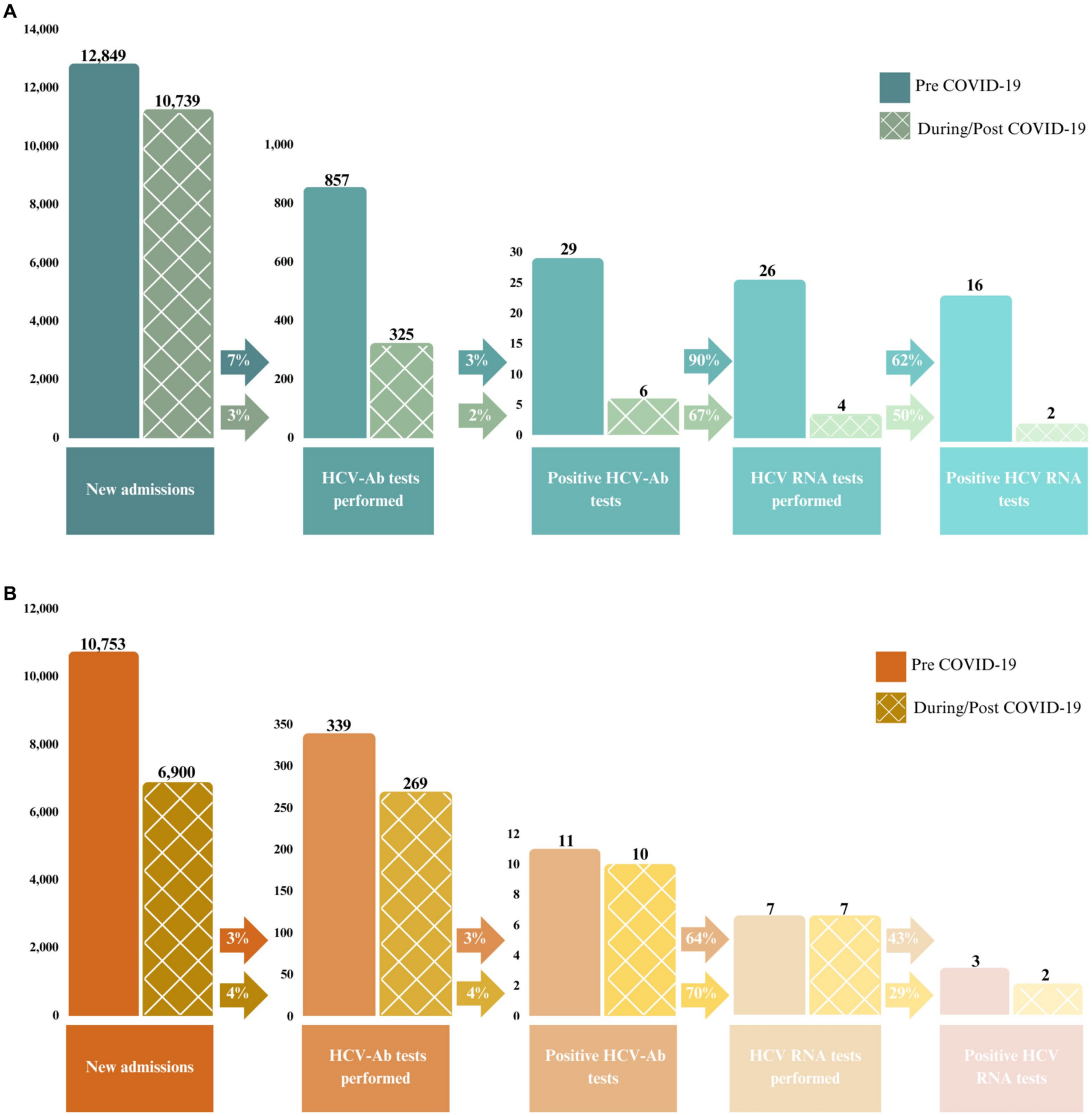
Number and proportion of positive HCV-Ab and HCV RNA tests performed between July 2018 and February 2022 at **(A)** EDM and **(B)** EDRDP. The solid bars represent the pre-COVID-19 pandemic period (July 2018 to February 2020) and the checkered bars represent the during/post COVID-19 pandemic period (March 2020 to February 2022).

Between July 2018 and February 2022, a total of 1,790 HCV-Ab tests were performed [*n* = 1,182 at EDM and *n* = 608 at EDRDP (Figure 2)]. Of these, there were 56 (3%) positive tests. There were 44 (79%) HCV RNA tests performed [30/35 (86%) at EDM and 14/21 (67%) at EDRDP]. Of these, just over half (23/44; 52%) were positive [18/30 (60%) at EDM and 5/14 (36%) at EDRDP].

Figure 2 illustrates the HCV care cascade among all new admissions up to positive HCV RNA at each prison, stratified into two time periods: pre-COVID-19 pandemic (prior to March 2020) and during/post COVID-19 pandemic (March 2020 onwards). Of note, at EDM, 7% of new admissions were screened for HCV-Ab using on-demand screening prior to the COVID-19 pandemic, which decreased to 3% of new admissions during/post COVID-19 pandemic. These percentages were 3 and 4%, respectively, at EDRDP. Similarly, the percentage of HCV-Ab positive individuals that underwent HCV RNA confirmatory testing decreased from 90% prior to the COVID-19 pandemic to 67% during/post COVID-19 pandemic at EDM, while these percentages were 64 and 70% at EDRDP, respectively.

**Figure 3:**
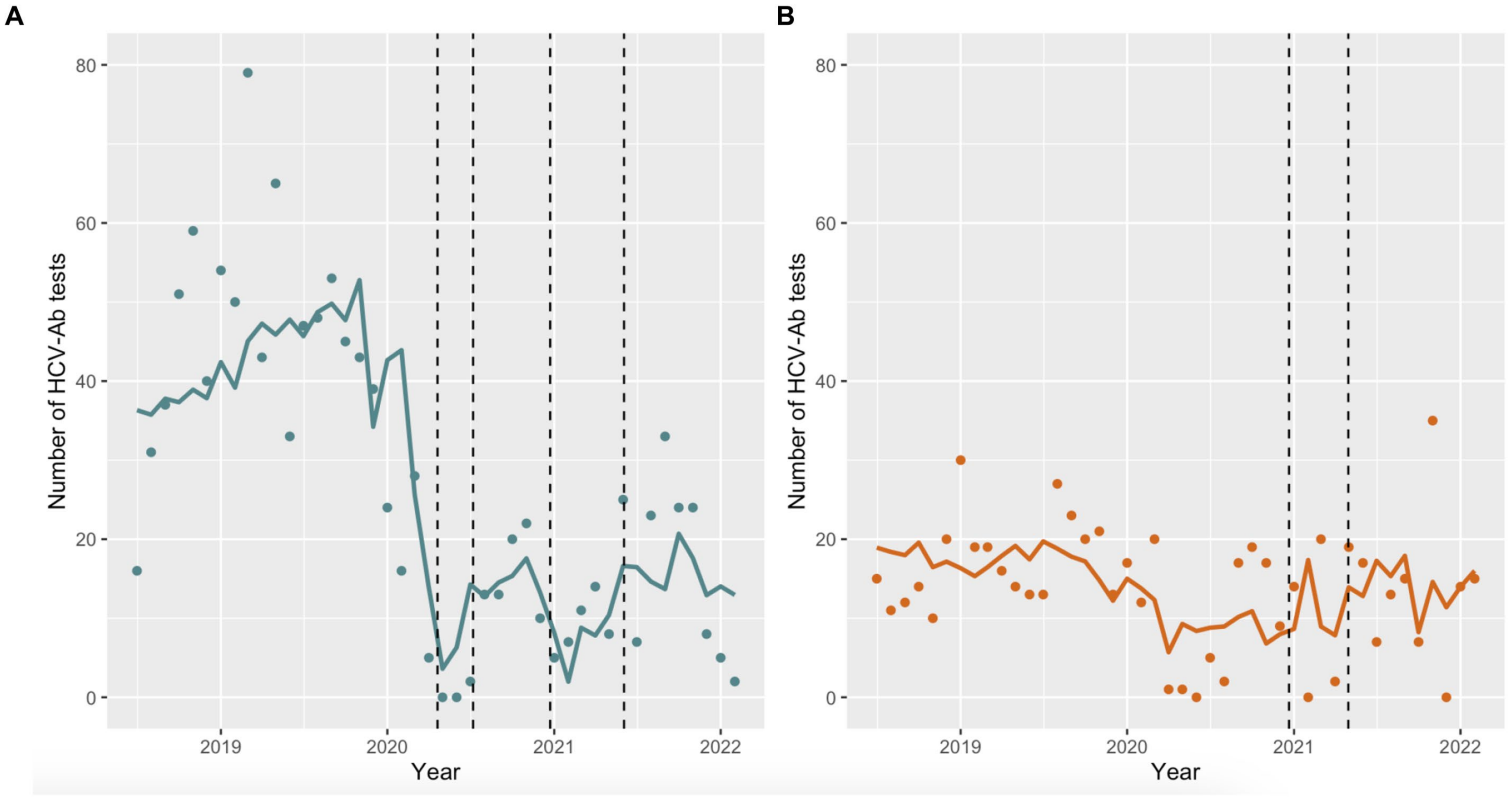
Predicted monthly HCV-Ab tests performed at each prison using negative binomial regression models. **(A)** The dashed lines represent the outbreaks at EDM (April 20 to July 7, 2020 and December 23, 2020 to June 3, 2021); **(B)** The dashed lines represent the outbreak at EDRDP (December 21, 2020 to April 30, 2021).

The number of HCV-Ab tests performed per month differed by prison and across time periods (Figures 3A,B). Prior to March 2020, the monthly number of HCV-Ab tests at EDM and EDRDP was approximately 40–50 and 15–20, respectively. During the first outbreak at EDM, the number of HCV-Ab tests performed at EDM per month decreased drastically (Figure 3A) – a trend that was maintained throughout the outbreak and post-outbreak periods. Figure 3B shows a slight decrease in the monthly number of HCV-Ab tests performed at EDRDP that coincides with the start of the first outbreak at EDM (April 2020) and continues until May 2021 (the end of the outbreak at EDRDP), after which HCV-Ab testing returned to pre-pandemic numbers.

Table 1 shows the estimates of the negative binomial regression, using the pre-outbreak period as the reference. There was a significant decrease in HCV-Ab screening at EDM during the outbreak (aOR 0.29; 95% CI 0.17–0.48) and post-outbreak (aOR 0.49; 95% CI 0.35–0.69) periods, compared to the pre-outbreak period. There was no significant change in HCV-Ab screening at EDRDP during the outbreak period (aOR 0.98; 95% CI 0.49–2.11) but a significant increase in HCV-Ab screening was observed post-outbreak (aOR 1.66; 95% CI 1.04–2.72), compared to the pre-outbreak period. Sensitivity analysis with a modified outbreak period showed similar trends for both prisons (results not shown).

**Table 1:** Estimated odds ratio of HCV-Ab screening by time periods and prisons.

Prison	Period	aOR (95% CI)*
EDM	Pre-outbreak	*Reference*
	Outbreak	0.29 (0.17–0.48)
	Post-outbreak	0.49 (0.35–0.69)
EDRPD	Pre-outbreak	*Reference*
	Outbreak	0.98 (0.49–2.11)
	Post-outbreak	1.66 (1.04–2.72)

*Number of new admissions per month was used as an offset.

95% CI, 95% confidence interval; aOR, adjusted odds ratio; EDM, l’Établissement de détention de Montréal; EDRDP, l’Établissement de détention de Rivière-des-Prairies.

## Discussion

A critical component of Canada’s HCV elimination strategy relies on active case finding of undiagnosed HCV (20), with carceral settings having been identified as crucial to case finding and thus, national HCV elimination efforts (21–24). We present the first study that evaluates the effect of the COVID-19 pandemic on HCV screening in any carceral setting worldwide. We found that HCV-Ab screening in the largest provincial prison in Quebec decreased significantly during the COVID-19 pandemic and had yet to rebound to pre-pandemic levels by the end of the study period. Conversely, the COVID-19 pandemic had minimal impact at a nearby remand prison with an increase in HCV-Ab screening post-outbreak to levels higher than pre-2020. To reach HCV elimination by 2030, attempts to minimize screening interruptions in carceral settings during future outbreaks should be prioritized, and screening for HCV during SARS-CoV-2 testing or vaccination could be considered (25, 26).

There are several reasons that may explain the expected decline in HCV screening in any carceral setting during the COVID-19 pandemic. These include decarceration (fewer people to screen), mandatory isolation periods on admission and following exposure to SARS-CoV-2 during incarceration (fewer opportunities to screen), and diverted financial and human resources (fewer resources for screening). Decarceration consists of the large-scale release of people who pose minimal risk to public safety, the increased use of home confinement, and the non-carceral management of people arrested for minor offenses (27). In Quebec, all these measures were put into place with explicit instructions to prioritize SARS-CoV-2 screening in provincial prisons as of April 2020, coinciding with the first large SARS-CoV-2 outbreak at EDM and increased nursing hours at each prison. It is therefore unsurprising that there was a significant decline in HCV-Ab testing during the outbreak period at EDM (despite controlling for the effect of decarceration) – the first prison in Quebec that experienced a SARS-CoV-2 outbreak affecting 95 incarcerated individuals (11). Through close observation, there was a similar, but less impressive decline in the number of HCV-Ab tests performed at EDRDP that coincided with EDM’s outbreak period – due to the presence of identical preventative measures at both prisons. It is thus possible that the impact of EDRDP’s outbreak on HCV-Ab screening was minimal since there had already been a decline in HCV-Ab testing preceding their outbreak (and mirroring EDM’s outbreak period).

Recovery of HCV screening post-pandemic has been inconsistent across Canada (8, 9). We found similar discrepancies in HCV-Ab screening in the post-outbreak periods across the two provincial prisons. HCV-Ab screening at EDM failed to recover to pre-pandemic levels while HCV-Ab screening at EDRDP was higher in the post-outbreak period compared to pre-2020. EDM experienced more frequent and larger outbreaks compared to EDRDP, possibly affecting the time to return to routine practices – that is, the impact of the outbreaks at EDM may have led to a reluctance to forego certain preventative measures in a timely manner, reflecting, to some degree, the inertia of the prison system. The failure to recover to pre-pandemic levels will likely have direct consequences on viral hepatitis elimination efforts (although not yet studied), and strategies to minimize screening interruptions in the event of future outbreaks should be explored. These include combination screening for HCV during SARS-CoV-2 viral testing, vaccination, or other health services, or the use of point-of care screening to accelerate engagement along the care cascade (28), both of which have been shown to be effective with other marginalized populations (25, 26). When faced with another pandemic, opportunistic screening – that is, the coupling of HCV testing with other health interventions (vaccination or other) that we might expect to continue in a pandemic – should be at the forefront of our elimination efforts.

Although STBBI screening in Quebec provincial prisons was re-prioritized in January 2021, differences in screening in the post-outbreak periods may also be explained by the divergent HCV screening strategies employed at each prison. On-demand screening has been shown to screen fewer individuals than risk-based screening (29), which may partially explain our findings. More importantly, the screening strategy at EDRDP changed to opt-in (from risk-based) in the post-outbreak period, explaining the significant increase in HCV-Ab tests compared to the pre-outbreak period. For HCV elimination to occur in Canadian provincial prisons, systematic screening (preferably via opt-out screening) should become standard of care (17–19, 22, 23, 30, 31).

There are limitations to our study. First, the available data was restricted to HCV tests obtained from two of the 16 provincial prisons in Quebec during the study period. As both prisons experienced different outbreaks, both in number and size, our results may not be generalizable to other prisons in Quebec or in Canada. Second, the implementation of emergency measures and the re-allocation of staff resources continuously changed throughout the COVID-19 pandemic, making it challenging to quantify these changes or their effects on HCV screening. Moreover, no record of the number of nursing hours dedicated to STBBI versus SARS-CoV-2 screening was available for either prison, resulting in raw estimates based on personal communications. Third, the number of monthly admissions is not equivalent to the number of unique individuals admitted each month, as the same individual can be re-incarcerated and counted multiple times in the number of new admissions over the study period. As such, the proportion of new entrants screened for HCV-Ab is likely under-estimated. Fourth, our study did not examine changes in the HCV care cascade distal to screening, including, but not limited to, linkage to HCV care and treatment uptake, the latter of which may have been impacted because each prison continues to assume all HCV treatment costs. Lastly, the limited sample size of RNA HCV testing precluded us from exploring changes in HCV RNA tests across time periods and by prisons.

In conclusion, the COVID-19 pandemic had a different impact on HCV screening at the two largest provincial prisons in Montreal, Quebec. Additional research is needed to understand these differences, but system-level inertia was likely an important factor in the failure to recover to post-pandemic levels. To eliminate HCV from carceral settings, attempts to minimize screening interruptions during future outbreaks should be prioritized, and combination screening for HCV and SARS-CoV-2 (with vaccination) could be considered.

## Data availability statement

The original contributions presented in the study are included in the article/supplementary material, further inquiries can be directed to the corresponding authors.

## Ethics statement

This study was approved by the McGill University Health Centre Research Ethics Board (MUHC REB #2022-8383). The studies were conducted in accordance with the local legislation and institutional requirements. Written informed consent for participation was not required from the participants or the participants’ legal guardians/next of kin in accordance with the national legislation and institutional requirements.

## Author contributions

NK: Conceptualization, Data curation, Formal analysis, Methodology, Supervision, Validation, Writing – original draft, Writing – review & editing. FL: Formal analysis, Methodology, Writing – original draft, Writing – review & editing. CD: Data curation, Formal analysis, Methodology, Writing – original draft, Writing – review & editing. GM: Data curation, Validation, Writing – review & editing. EG: Data curation, Validation, Writing – review & editing. MP: Writing – review & editing. JC: Writing – review & editing.
